# Temporal and periorbital depressions identified by 3D images are correlated with malnutrition phenotypes in cancer patients: A pilot study

**DOI:** 10.3389/fnut.2023.1115079

**Published:** 2023-03-13

**Authors:** Moxi Chen, Xue Wang, Meifen Han, Yunzhu Li, Nanze Yu, Xiao Long, Wei Chen

**Affiliations:** ^1^Department of Clinical Nutrition, Peking Union Medical College Hospital, Chinese Academy of Medical Sciences & Peking Union Medical College, Beijing, China; ^2^Department of Pharmacy, Peking Union Medical College Hospital, Chinese Academy of Medical Science & Peking Union Medical College, Beijing, China; ^3^School of Basic Medicine and Clinical Pharmacy, China Pharmaceutical University, Nanjing, China; ^4^Department of Plastic Surgery, Peking Union Medical College Hospital, Beijing, China

**Keywords:** cancer, three-dimensional images, temporal muscle, phenotype, malnutrition

## Abstract

**Background:**

Prompt diagnosis of malnutrition and appropriate interventions can substantially improve the prognosis of patients with cancer; however, it is difficult to unify the tools for screening malnutrition risk. 3D imaging technology has been emerging as an approach to assisting in the diagnosis of diseases, and we designed this study to explore its application value in identifying the malnutrition phenotype and evaluating nutrition status.

**Methods:**

Hospitalized patients treating with maintenance chemotherapy for advanced malignant tumor of digestive system were recruited from the Department of Oncology, whose NRS 2002 score > 3. Physical examination and body composition data of patients at risk for malnutrition were analyzed by physicians trained to complete a subjective global assessment. The facial depression index was recognized using the Antera 3D® system, temporal and periorbital depression indexes were acquired using the companion software Antera Pro. This software captures quantitative data of depression volume, affected area, and maximum depth of temporal and periorbital concave areas.

**Results:**

A total of 53 inpatients with malnutrition-related indicators were included. The volume of temporal depression was significantly negatively correlated with upper arm circumference (*r* = −0.293, *p* = 0.033) and calf circumference (*r* = −0.285, *p* = 0.038). The volume and affected area of periorbital depression were significantly negatively correlated with fat mass index (*r* = −0.273, *p* = 0.048 and *r* = −0.304, *p* = 0.026, respectively) and percent body fat (*r* = −0.317, *p* = 0.021 and *r* = −0.364, *p* = 0.007, respectively). The volume and affected area of temporal depression in patients with muscle loss phenotype (low arm circumference/low calf circumference/low handgrip strength/low fat-free mass index) were significantly higher than those in patients without muscle loss. Moreover, patients with fat mass loss phenotype (low fat mass index) showed a significant increase in the volume and affected area of periorbital depression.

**Conclusion:**

The facial temporal region, and periorbital depression indicators extracted by 3D image recognition technology were significantly associated with the phenotype of malnutrition-related muscle and fat loss and showed a trend of grade changes in the population of different subjective global assessment nutritional classifications.

## 1. Introduction

Patients with cancer usually have the highest incidence of malnutrition among hospitalized patients as both the disease and treatment can lead to alterations in energy expenditure and energy intake (1). Malnutrition reduces sensitivity to drugs, decreases quality of life, and directly increases the risk of all-cause mortality (2). Prompt diagnosis of malnutrition and appropriate interventions can significantly improve the prognosis of patients with cancer (3). Therefore, screening for malnutrition risk at admission is recommended for all patients with cancer; however, screening tools differ between regions and organizations.

In the past few decades, screening for malnutrition including a comprehensive assessment of the patients’ weight changes, food intake, and functional levels has mainly been performed through subjective assessments by clinicians and nutritionists (4). Due to the differences in target populations and evaluation methods, it is challenging to standardize the evaluation of the malnutrition status of patients. In recent years, associations such as the European Society for Clinical Nutrition and Metabolism and American Society for Parenteral and Enteral Nutrition (ASPEN) have reached agreement on the application of dual-energy X-ray absorptiometry (DXA), computed tomography (CT), magnetic resonance imaging (MRI), and other objective assessment indicators for quantifying the muscle and fat mass for diagnosis of malnutrition (5). Through extensive clinical applications, cut-off values of these indicators have been established in various populations. The clinical value of muscle mass and fat mass assessments in the diagnosis of malnutrition has gradually received increasing attention.

The psoas index, quantified by CT imaging of the third lumbar spine (L3), has been recognized as an important indicator of muscle loss, and is significantly associated with morbidity and long-term prognosis in patients with cancer (6). A comprehensive assessment of the changes in body composition at different anatomical locations (carina, thoracic, and lumbar spine) using multiple levels of CT imaging has also been found to be useful in predicting the prognosis of lung transplant patients (7). However, because DXA, CT, and MRI are not widely used in the assessment of malnutrition in clinical practice, and due to their relatively high cost, some simple and available surrogate indicators are used to evaluate muscle loss, such as the circumference of the upper arm and calf and bioelectrical impedance analysis (BIA) of body composition (8).

In a recent study, temporal muscle thickness (TMT) assessed using 3D imaging technology was used as a quantitative muscle biomarker for predicting progression-free survival and overall survival in patients with primary diffuse large B-cell lymphoma of the central nervous system (PCNSL) (9). This study demonstrated the utility of computer-assisted image recognition techniques for accurate measurement of body composition. 3D image recognition is an advanced technology that uses computer programs to assist with identification of image features. This technology can discern subtle changes that are difficult to detect by visual examination alone, and can integrate the characteristics of many variables for computational analysis. It has been widely used in recent years to assist in clinical diagnosis.

3D facial image recognition has demonstrated its clinical application in the analysis of typical facial features of acromegaly (10), differential diagnosis of various genetic syndromes involving facial deformities (11), and diagnosis and analysis of treatment efficacy of skin lesions (12). Moreover, the simplicity of capturing 3D facial images using mobile phone applications makes this auxiliary diagnosis method more accessible, and therefore more conducive in various clinical and preclinical settings.

To explore the application value of 3D facial image recognition technology in assisting the diagnosis of malnutrition, we piloted a 3D facial image recognition method for hospitalized patients with cancer. We aimed to verify its applicability in determining the phenotype related to malnutrition, using measures such as the reduction in muscle and fat mass, and thus provided a methodological reference for simplifying the clinical diagnosis of malnutrition.

## 2. Methods

### 2.1. Study population

Hospitalized patients treating with maintenance chemotherapy for advanced malignant tumor of digestive system were recruited from the Department of Oncology, Peking Union Medical College Hospital. The inclusion criteria were: (1) Han race, (2) age > 18 years, (3) nutritional risk screening (NRS 2002) score > 3, and (4) voluntary participation and cooperation for facial image collection. The exclusion criteria were: (1) artificial changes in the face (for example, facial plastic surgery or trauma, head and neck radiotherapy); (2) special diseases with facial changes (for example, acromegaly, hyperthyroidism); (3) administration of high-dose glucocorticoids leading to facial changes; (4) edema of the face or limbs; and (5) other situations deemed for exclusion by the researchers. This study was approved by the Ethics Committee of the Peking Union Medical College Hospital (Number: JS-2768), and all participants provided written informed consent.

### 2.2. Malnutrition phenotypic assessment

Prior to the start of the trial, two physicians were uniformly trained in the content and standard procedures of nutritional assessments, and any doubts and inconsistencies were discussed and resolved. Physicians were trained to complete NRS 2002 and subjective global assessment (SGA) nutritional assessment by applying structured questionnaires within 24 h of patient admission and conducting a standardized physical examination, which included measurement of height, weight, upper arm circumference, calf circumference, handgrip strength (HGS), and body composition analysis.

NRS 2002 and SGAs were performed in accordance with the guidelines of the American Society for Parenteral and Enteral Nutrition and were derived from the patient’s medical history and physical examination. The medical history included weight, appetite changes, gastrointestinal symptoms, mobility, and disease-related nutritional requirements over the past 2 weeks. Physical examination included subcutaneous fat (triceps and chest), muscle mass (including the quadriceps and deltoid), and edema levels. Based on the above assessments, patients were classified into grades A, B, and C, with grades B and C considered to have mild-to-moderate and severe malnutrition, respectively.

Weight (kg) and height (cm) were measured in light indoor clothing without shoes. Body mass index (BMI) was calculated as weight (kg) divided by height (m) squared (kg/m^2^). The upper arm circumference (AC) and calf circumference (CC) were measured using a non-elastic tape, with a minimum reading of 0.1 cm. With the patient in a sitting position, the circumference of the relaxed non-dominant arm at the midpoint of the line connecting the acromion and olecranon was measured. Additionally, with the patient sitting on the side of the bed, the relaxed calf was measured on a plane perpendicular to the long axis of the calf to obtain the maximum circumferential value. HGS was measured using an electronic handgrip dynamometer with the patient standing comfortably, repeated three times with the lateral hand; the maximum value was used for the analysis.

Body composition analysis of the patients was performed using BIA in a supine position with arms held away from the body and legs apart. When the patients were unable to take the supine position, the sitting position was used for BIA measurement (only two of all the subjects). Fat-free mass (FFM), fat mass (FAT), visceral fat mass (VFA), and percent body fat (PBF) were measured using a portable body composition meter (InBody S10); the fat-free mass index (FFMI) and fat mass index (FMI) were calculated as FFM and FAT (kg) divided by height (m), respectively.

### 2.3. 3D facial image recognition

Facial depression index recognition was performed using the Antera 3D® system within 24 h of patient admission, using a camera for acquisition and analysis of skin images, with an area of 56 mm × 56 mm. The camera utilizes light-emitting diodes (LEDs) of different wavelengths to illuminate the skin from different directions and then performs computer-assisted 3D skin surface reconstruction based on spatial and spectral analysis of the acquired image data (13). The reconstructed skin texture using the shape from shading technique was used for quantitative skin analysis, and different filters (14) were available for the measurement of specific skin features such as wrinkles, texture, pores, depressions, and elevations. Volume-related measurements were generated by interpolating the information from the boundaries of the selected region to determine the enclosed volume. The camera opening was placed directly on the skin, and the image was unaffected by external lighting conditions. This was achieved through a combination of polarizing filters and proprietary technology, ensuring that the results were accurate and reproducible (15).

**Figure 1:**
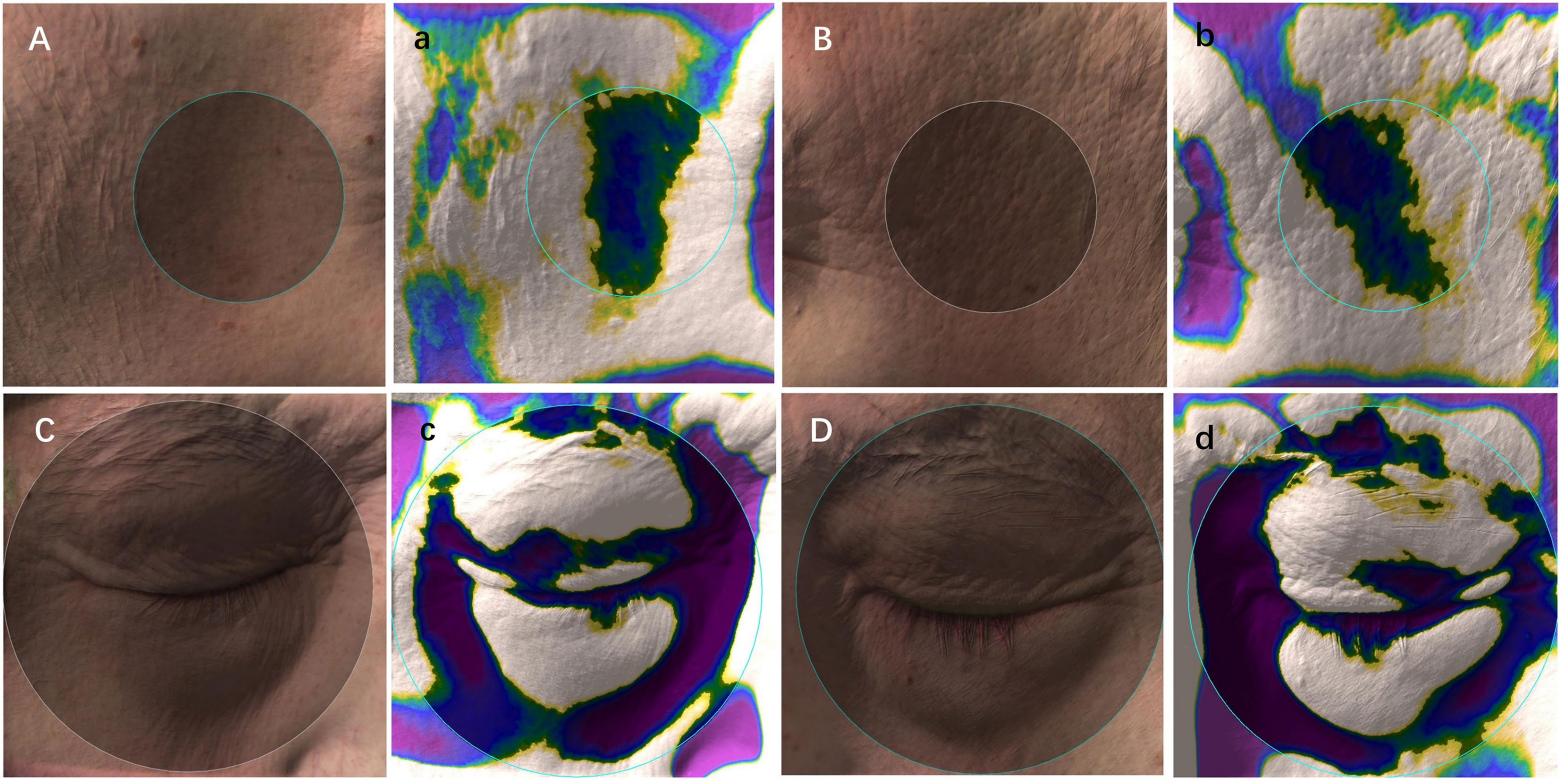
Schematic diagram of 3D image recognition and depression index acquisition. **(A–D)**: skin images; **(a–d)**: depression index sampling (gray region, the reference level; green, blue, and purple regions, depressions); Note: the circle indicated the pre-specified target area to obtain the quantitative data of depression volume, affected area, and maximum depth of temporal (diameter = 30.8 mm) and periorbital (diameter = 54.1 mm).

Depression index of temporal (between the outer edge of the orbit and the hairline) and periorbital (the upper edge was the brow arch, covering the entire orbit) were acquired using the camera companion software Antera Pro (v2.8.2; Miravex Limited, Dublin, Ireland) (16). This software analyzed the skin texture of the pre-specified target area (area indicated by the circle in Figure 1), and obtained the quantitative data of depression volume, affected area, and maximum depth of temporal (TEM) (diameter = 30.8 mm) and periorbital (ORB) (diameter = 54.1 mm) concave areas (purple, blue, and green regions in Figure 1; gray region represents the reference level).

### 2.4. Malnutrition phenotype groups

To compare differences in depression indicators among different groups of malnutrition phenotype, the phenotypes were grouped according to the results of previous studies and guidelines. Among the phenotypes associated with decreased muscle mass, upper arm circumference (AC) cutoffs were <27 cm in men and <25 cm in women (17). and calf circumference (CC) cutoff was <33 cm in men and <32 cm in women (18). According to the 2019 Consensus Update on Sarcopenia Diagnosis and Treatment developed by the Asian Working Group for Sarcopenia, the criteria for low HGS are <28 kg in men and <18 kg in women (19); and the cut-off FFMI calculated from body composition measurements is <17 kg/m^2^ in men and <15 kg/m^2^ in women. Malnutrition phenotypes associated with reduced fat mass were FMI with cut-off value of <7.7 kg/m^2^ in men and <5 kg/m^2^ in women (20).

### 2.5. Statistical analysis

Previous studies have shown that the incidence of malnutrition in hospitalized patients with cancer in China was approximately 38.9% (21); the type I error α was relaxed to 0.25 in a pilot study (22), and the permissible error δ was set at 0.078. Thus, at least 52 patients had to be included in this study. All statistical analysis was performed using the SPSS statistical software version 22.0 (IBM Corp., Armonk, NY, United States). Continuous variables were expressed as the mean ± standard deviation. The Shapiro–Wilk tests were used to assess normal distribution of variables, variables with non-normal distribution were expressed as median (IQR). One-way ANOVA and Kruskal–Wallis H-test were used to compare the differences of malnutrition phenotypes and depression indices among the SGA groups. The independent samples *t*-test and Mann–Whitney U-test were used to compare the differences of depression between different groups of malnutrition phenotypes. Categorical variables were described as frequencies and percentages, and comparisons between groups were performed using the chi-square and Fisher test. Spearman correlation analysis was used to test the association between depression index and malnutrition phenotype. All tests were two-sided, and statistical significance was set at *p* < 0.05.

## 3. Results

### 3.1. Characteristics related to malnutrition phenotypes

Data of 53 inpatients with malnutrition indicators were successfully collected in this pilot study. According to the SGA assessment, 30 patients had no risk of malnutrition (SGA A), 15 had mild-to-moderate malnutrition (SGA B), and 8 had severe malnutrition (SGA C). The distribution of clinical characteristics and malnutrition-related phenotypes among groups are presented in Table 1. Age, sex, and tumor location were evenly distributed among theree groups. In addition to most of the tumors located in the gastrointestinal tract (74%), a few patients were in the liver (4%), biliary tract (4%), pancreas (4%), etc. Except for HGS, FFMI, and VFM, measured malnutrition phenotypes (including BMI, AC, CC from physical examination, and FAT, FMI, and PBF measured by body composition analysis) were significantly different, and showed a downward trend along with the grade of malnutrition.

**Table 1:** Basic characteristics of malnutrition phenotypes in the study population.

Phenotypic characteristics	SGA A (*N* = 30)	SGA B (*N* = 15)	SGA C (*N* = 8)	*p*-value
	Mean ± SD /Median(IQR)	Mean ± SD /Median(IQR)	Mean ± SD /Median(IQR)	
Age (years)	57.70 ± 10.67	62.47 ± 9.32	55.25 ± 13.01	0.237
Sex (male, %)	18 (60)	13 (87)	6 (75)	0.197
Tumor location (gastrointestinal tract, %)	21 (70)	12 (80)	6 (75)	0.907
BMI (kg/m^2^)	24.12 ± 2.99	23.07 ± 2.86	18.84 ± 2.69	<0.001^***^
AC (cm)	28.50 (4.10)	29.00 (4.00)	24.50 (3.00)	0.006^**^
CC (cm)	36.95 ± 2.26	35.58 ± 3.21	32.73 ± 4.38	0.003^**^
HGS (kg)	26.75 ± 7.64	28.77 ± 6.83	29.5 ± 11.35	0.587
FFMI (kg/m)	18.44 ± 2.30	18.49 ± 1.66	16.37 ± 2.45	0.051
FAT (kg)	16.05 ± 7.19	13.35 ± 6.33	6.99 ± 2.07	0.004^**^
FMI (kg/m)	5.68 ± 2.62	4.59 ± 2.08	2.47 ± 0.70	0.003^**^
VFA (kg)	54.15 (54.50)	42.00 (49.50)	31.20 (7.00)	0.072
PBF (%)	23.03 ± 9.14	19.29 ± 6.98	13.09 ± 3.25	0.009^**^

One-way ANOVA, Kruskal–Wallis H-test (AC, VFA), Fisher test (Sex, Tumor location): *p < 0.05, **p < 0.01, ***p < 0.001. AC, arm circumference; CC, calf circumference; FAT, fat mass; FFMI, fat-free mass index; FMI, fat mass index; HGS, hand grip strength; PBF, percent body fat; VFA, visceral fat mass.

### 3.2. 3D facial image recognition depression indicators and SGA

In the temporal region, the volume (SGA A: 71.05 ± 27.20 mm^3^, SGA B: 75.59 ± 31.72 mm^3^, SGA C: 99.66 ± 38.34 mm^3^, *p* = 0.068) and surface area (SGA A: 244.71 ± 63.41 mm^2^, SGA B: 259.21 ± 74.59 mm^2^, SGA C: 315.10 ± 84.66 mm^2^, *p* = 0.049) of depression showed a significant grading trend among dystrophic groups; the more severe the degree of malnutrition, the larger the volume or surface area of depression. No significant differences were found among the groups with respect to the maximum depth of depression (Table 2).

**Table 2:** Depression index differences among malnourished groups.

Depression index	SGA A (*N* = 30)	SGA B (*N* = 15)	SGA C (*N* = 8)	*p*-value
	Mean ± SD /Median(IQR)	Mean ± SD /Median(IQR)	Mean ± SD /Median(IQR)	
TEM-volume (mm^3^)	71.05 ± 27.20	75.59 ± 31.72	99.66 ± 38.34	0.068
TEM-area (mm^2^)	244.71 ± 63.41	259.21 ± 74.59	315.10 ± 84.66	0.049^*^
TEM-depth (mm)	0.60 (0.42)	0.56 (0.52)	0.68 (0.47)	0.760
ORB-volume (mm^3^)	703.37 ± 145.48	784.73 ± 160.57	737.88 ± 140.92	0.234
ORB-area (mm^2^)	1,061.09 ± 104.79	1,102.53 ± 98.07	1,108.63 ± 98.95	0.312
ORB-depth (mm)	2.46 ± 0.49	2.56 ± 0.40	2.39 ± 0.35	0.641

One-way ANOVA, Kruskal–Wallis H-test (TEM-depth): *p < 0.05, **p < 0.01, ***p < 0.001. ORB, periorbital region; TEM, temporal region.

In the periorbital region, the surface area of periorbital depressions also showed a significant grading trend among the three groups. Patients in the SGA A group had the minimum affected area (1,061.09 ± 104.79 mm^2^), followed by the SGA B group (1,102.53 ± 98.07 mm^2^), while SGA C patients showed the largest depression (1,108.63 ± 98.95 mm^2^). No obvious trends were found in the volume or maximum depth of periorbital depression among the groups (Table 2).

### 3.3. 3D facial image recognition depression indicators and malnutrition phenotypes

**Figure 2:**
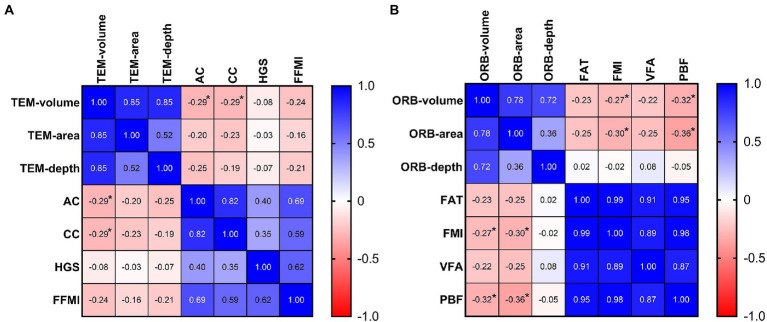
The correlation heatmaps between depression index and malnutrition phenotype. **(A)** The negative correlation between the temporal depression volume and muscle mass phenotype (AC and CC). **(B)** The negative correlation between the periorbital depression index (volume and surface area) and fat mass phenotype (FMI and PBF). AC, arm circumference; CC, calf circumference; FAT, fat mass; FFMI, fat-free mass index; FMI, fat mass index; HGS, hand grip strength; ORB, periorbital region; PBF, percent body fat; TEM, temporal region; VFA, visceral fat mass. Spearman correlation analysis: *p < 0.05.

The volume of temporal depression was significantly negatively correlated with the upper AC (*r* = −0.293, *p* = 0.033) and CC (*r* = −0.285, *p* = 0.038). A larger upper AC or CC implies higher muscle mass and was found to be related to smaller volume of temporal depressions, while the phenotypes related to muscle mass, including HGS and FFMI, also exhibited a negative correlation with measures of temporal depression; however, the result was not statistically significant (Figure 2A).

The volume and surface area of the periorbital depressions were significantly negatively correlated with the FMI (volume: *r* = −0.273, *p* = 0.048; surface area: *r* = −0.304, *p* = 0.026) and PBF (volume: *r* = −0.317, *p* = 0.021; surface area: *r* = −0.364, *p* = 0.007). Both FMI and PBF are indicators of body fat content, and patients with more body fat exhibited smaller volume and surface area of periorbital depression. Moreover, FAT and VFM showed a negative correlation with the indices of periorbital depression; however, the difference was not statistically significant (Figure 2B).

Among the groups categorized based on the indicators of malnutrition phenotype, the volume and surface area of temporal depression in patients with muscle loss phenotypes (low AC/low CC/low HGS/low FFMI) were significantly higher than those in patients without muscle loss. Patients with the fat mass loss phenotype (low FMI) also showed a significant increase in the volume and surface area of periorbital depression. The maximum depth of temporal and periorbital depression showed consistent trends between the groups; however, it was not statistically significant (Table 3).

**Table 3:** Depression index differences among various malnutrition phenotype groups.

Phenotype	Group (number)	TEM-volume (mm^3^)		TEM-area (mm^2^)		TEM-depth (mm)	
		Mean ± SD/Median (IQR)	*p*-value	Mean ± SD/Median (IQR)	*p*-value	Median (IQR)	*p*-value
AC (cm)	*M* < 27, *F* < 25 (14)	93.92 ± 31.11	0.015^*^	300.95 ± 74.49	0.011^*^	0.68 (0.46)	0.204
	*M* > 27, *F* > 25 (39)	70.45 ± 29.36		244.53 ± 67.09		0.58 (0.48)	
CC (cm)	*M* < 33, *F* < 32 (7)	100.26 ± 32.16	0.031^*^	358.51 (127.51)	0.024^*^	0.67 (0.45)	0.546
	*M* > 33, *F* > 32 (46)	73.06 ± 29.93		242.14 (102.98)		0.60 (0.42)	
HGS (kg)	*M* < 28, *F* < 18 (16)	89.17 ± 31.58	0.055	289.2 ± 65.88	0.049^*^	0.72 (0.49)	0.215
	*M* > 28, *F* > 18 (37)	71.24 ± 30.03		246.57 ± 72.76		0.60 (0.41)	
FFMI (kg/m)	M < 17, *F* < 15 (10)	103.13 ± 35.03	0.002^**^	313.4 ± 84.09	0.008^**^	0.87 (0.47)	0.059
	M > 17, *F* > 15 (43)	70.49 ± 27.32		246.89 ± 64.81		0.58 (0.33)	
Phenotype	Group (number)	ORB-volume (mm^3^)		ORB-area (mm^2^)		ORB-depth (mm)	
		Mean ± SD	*p*-value	Mean ± SD	*p*-value	Mean ± SD	*p*-value
FMI (kg/m)	M < 7.7, *F* < 5 (14)	761.37 ± 156.36	0.003^**^	1,106.34 ± 100.56	0.001^**^	2.48 ± 0.47	0.977
	M > 7.7, *F* > 5 (39)	648.69 ± 96.25		1,006.60 ± 67.83		2.47 ± 0.40	

Independent sample *t*-test, Mann–Whitney U-test (CC&TEM-area, TEM-depth): *p < 0.05, **p < 0.01, ***p < 0.001. AC, arm circumference; CC, calf circumference; FFMI, fat-free mass index; FMI, fat mass index; HGS, hand grip strength; ORB, periorbital region; TEM, temporal region.

## 4. Discussion

Our study has provided further evidence to support the association between face-specific depression indicators and dystrophic phenotypes. In particular, the degree of temporal depression was inversely correlated with the level of body muscle mass, and the degree of periorbital depression was negatively correlated with the level of subcutaneous fat mass. These findings were consistent with the results of nutritional physical examinations and suggest the application value of 3D image recognition of facial features in the diagnosis of malnutrition.

In our study, malnutrition-related phenotypes (anthropometric measures of muscle and fat mass) showed significant differences among patients with or without malnutrition; however, HGS, an indicator of muscle function, did not vary among groups. This indicates that although patients with cancer are at risk of muscle loss because of the inflammatory burden of tumor, they can still maintain a certain degree of muscle quality with active treatment and functional training (23), and thus reduce the adverse effects of malnutrition. Moreover, the differences between manually measured phenotypic indicators and objective indicators measured by body composition analysis suggest possible sensitivity differences between subjective malnutrition assessment and objective nutritional status (24), indicating the importance of a standardized malnutrition diagnosis.

In the nutrition-focused physical assessment (NFPA) proposed by the American Academy of Nutrition and Dietetics and ASPEN (25), the amount of temporal muscles was identified as an evaluation indicator for changes in body muscle mass. Recent studies have confirmed the association between temporal muscle thickness (measured using ultrasound (26) or CT (27)) and muscle mass, energy expenditure (28), or nutritional status. This is consistent with our study findings, indicating an association between temporal muscle atrophy-related depression indices and body muscle loss or malnutrition. However, our study did not find the correlation between temporal depressions and handgrip strengths, which might be due to the inconsistency between muscle quality and muscle mass in our participants, and suggested that the function of upper limb muscle may not be related to the volume of facial muscle.

Moreover, the NFPA guidelines use the periorbital fat pad as an important phenotypic indicator to evaluate the level of subcutaneous fat. Our study confirmed the consistency between the degree of periorbital depression and change in body fat mass, However, the thickness of the periorbital fat pad was also affected by age (29), sex (30), diseases (31), and other factors, which may explain the reason for no significant differences being observed in the periorbital depression indicators among patients with malnutrition of different grades. Our study also showed that the decrease of body fat had limited effect on the depth of periorbital depression, and the volume and surface area of periorbital depression should be taken into account when evaluating the periorbital fat pad. In addition, our study indicated that periorbital depressions mainly reflected changes in subcutaneous fat mass, and its relationship with VFM requires further research.

Computer-assisted image recognition technology has been widely used in many diseases, including cardiovascular diseases (32), digestive system diseases (33), and skin lesions (34), 3D facial imaging can not only quantify facial features comprehensively and accurately but is also favored by many mobile phone programs because of convenient sampling and simplified technical difficulty (35). As a multi-system and multi-dimensional assessment, malnutrition diagnosis has no unified and standard evaluation system. Thus, the promotion of malnutrition diagnosis in the clinical environment requires high cost on personnel training, and it is difficult to achieve consistency. In the future, the use of simple and fast 3D image recognition technology to obtain malnutrition characteristics, combined with machine learning technology to accurately identify malnourished patients, will help in early screening of malnutrition risk in preclinical settings and timely provision of interventions to improve its prognosis. This is one of the innovative ways for malnutrition assessment system to be effectively implemented in grass-roots medical and health institutions, such as community hospitals, nursing institutions and mental illness centers.

Our study also has some limitations. First, being a pilot study, the sample size was small; however, distribution of malnutrition among the included patients was consistent with the prevalence of malnutrition in hospitalized patients with cancer found in previous surveys, indicating that the study population was still representative to some extent. Second, we did not use the gold standard DXA, CT, and MRI for muscle mass measurement but instead used portable BIA, which reduced the accuracy of body composition measurement. However, BIA is more widely used in clinical settings, and the cost is relatively low, which is conducive to the promotion and verification of tests in a large population. Finally, the 3D image analysis method used in this study is relatively simple, and the accuracy and quantity of extracted data from facial features are limited, which may be the reason why we found that the correlation between facial depressions and malnutrition phenotype is relatively low (0.2–0.4). In a later study with an enlarged amount of facial image data, more image recognition and machine learning technologies will be introduced to improve the accuracy of facial recognition and malnutrition diagnosis, and to further verify the effectiveness of 3D facial image in the diagnosis of malnutrition.

## 5. Conclusion

The facial temporal region and periorbital depression indicators extracted by 3D image recognition technology were significantly associated with the phenotype of malnutrition-related muscle loss and fat loss and showed a trend of grade changes in the population of different SGA nutritional classifications. 3D facial image recognition technology is expected to become an important clinical auxiliary tool for extracting phenotypic indicators of malnutrition in the population and for early warning of malnutrition risk.

## Data availability statement

The raw data supporting the conclusions of this article will be made available by the authors, without undue reservation.

## Ethics statement

The studies involving human participants were reviewed and approved by Ethics Committee of the Peking Union Medical College Hospital (Number: JS-2768). The patients/participants provided their written informed consent to participate in this study. Written informed consent was obtained from the individual (s) for the publication of any potentially identifiable images or data included in this article.

## Author contributions

XL and WC equally contributed to the conception and design of the research. XW and MH contributed to the acquisition of the data. YL and NY contributed to the interpretation of the data. MC analyzed the data and drafted the manuscript. All authors critically revised the manuscript, agree to be fully accountable for ensuring the integrity and accuracy of the work, and have read and approved this statement.

## Funding

This work was supported by the Grants from National Natural Science Foundation of China (grant number: 72074222).

## Conflict of interest

The authors declare that the research was conducted in the absence of any commercial or financial relationships that could be construed as a potential conflict of interest.

## Publisher’s note

All claims expressed in this article are solely those of the authors and do not necessarily represent those of their affiliated organizations, or those of the publisher, the editors and the reviewers. Any product that may be evaluated in this article, or claim that may be made by its manufacturer, is not guaranteed or endorsed by the publisher.
